# Plasma cell myeloma in a 9‐year‐old male: Case report and literature review

**DOI:** 10.1002/ccr3.8801

**Published:** 2024-04-20

**Authors:** Kato Ronald, Ambaru Jacinta, Ssebagala Umaru

**Affiliations:** ^1^ Department of Emergency Medicine Savannah Hospital Nairobi Kenya; ^2^ Department of Oncology Uganda Cancer Institute Kampala Uganda; ^3^ Department of Internal Medicine King David Hospital Nairobi Kenya

**Keywords:** biopsy, histology, immunoglobulin, lesion, plasma cell myeloma

## Abstract

**Key Clinical Message:**

Plasma cell myeloma is a rare entity in the pediatric population. The peak incidence is in the seventh decade, with less than 2% of cases occurring in patients under the age of 40. It is worth noting that any destructive bony lesion in a child should be investigated.

**Abstract:**

Plasma cell myeloma (multiple myeloma) is the most common form of plasma cell neoplasm. It is a rare entity in young patients. The peak incidence is in the seventh decade, with less than 2% of cases occurring in patients under the age of 40. A male patient aged 9 years old with a progressive pain in lower back for about 1 month, aggravated by bending, associated with inability to stand upright, no any history of trauma. He complained about left pin‐point chest pain, no any history of febrile illness. MRI showed a mass lesion of the L3 vertebra; CT scan revealed osteolytic lesions in the left T12, S2‐sacral region, and left calvarium. Histology report of L3 lesion revealed cells with an eccentric nucleus, prominent Golgi apparatus and Flow cytometry revealed cells stained positive for CD 138 and CD56 and negative for CD45 expression. In situ hybridization identified k‐light chain band restriction. Bone marrow evaluation was normal. A small serum monoclonal immunoglobin A spike of k‐light chain type was noted. Other tests like complete blood count, lactate dehydrogenase levels, renal functional tests, and B2‐microglobulin were normal. A diagnosis of plasma cell myeloma was made and the patient was started on emergent radiation to L3 lesion due to progressive neurological symptoms followed systemic therapy which resulted int reduction of L3 lesion. Plasma cell myeloma is extremely rare form of liquid tumor in the pediatric population, and it is important for any destructive bony lesion in a child to have appropriate work up.

## 1 INTRODUCTION

Plasma cell myeloma (multiple myeloma) is the most common form of plasma cell neoplasm. The incidence of myeloma increases with age; incidence has increased over 40% in the United States since 1990, while global mortality has risen by 94% and mortality has fallen by 18%. The 5 year survival is more than doubled over the past decades with the new modalities. Risk factors include age with average age of diagnosis is 69, race basically in African Americans are over double as likely to be diagnosed, with male at a 1.5× risk, and family history. It is extremely rare in the pediatric age group. While a number of cases have been reported that lacked convincing evidence for the diagnosis, only two reports of well‐documented patients with myeloma have been described younger than age 30 years.^1^

## 2 CASE REPORT

### 2.1 History and examination

Patient presented with a progressive pain in lower back for about 1 month, aggravated by bending, associated with inability to stand upright, no any history of trauma. He complained about left pin‐point chest pain, no any history of febrile illness.

### 2.2 Methods

#### 2.2.1 Differential diagnosis

1. Pediatric spinal cord tumor.
2. Chronic osteomyelitis.

#### 2.2.2 Investigations and treatment

On examination, he was sick looking, with lower limb paresthesia and reduced muscle power and bulk.

**FIGURE 1:**
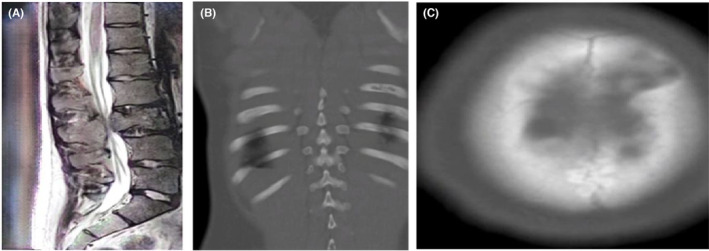
(A) MRI showing a destructive lesion at L3 vertebra. (B) CT‐scan chest showing osteolytic lesion in the left third Rib. (C) CT‐scan head showing osteolytic lesion in left parasagittal calvarium.

The patient underwent several work‐ups which included, lumber spine MRI revealed a distorted lesion of L3 (Figure 1A), CT‐scan chest revealed osteolytic lesions in the left third rib (Figure 1B), and CT scan of the left calvarium (Figure 1C).

**FIGURE 2:**
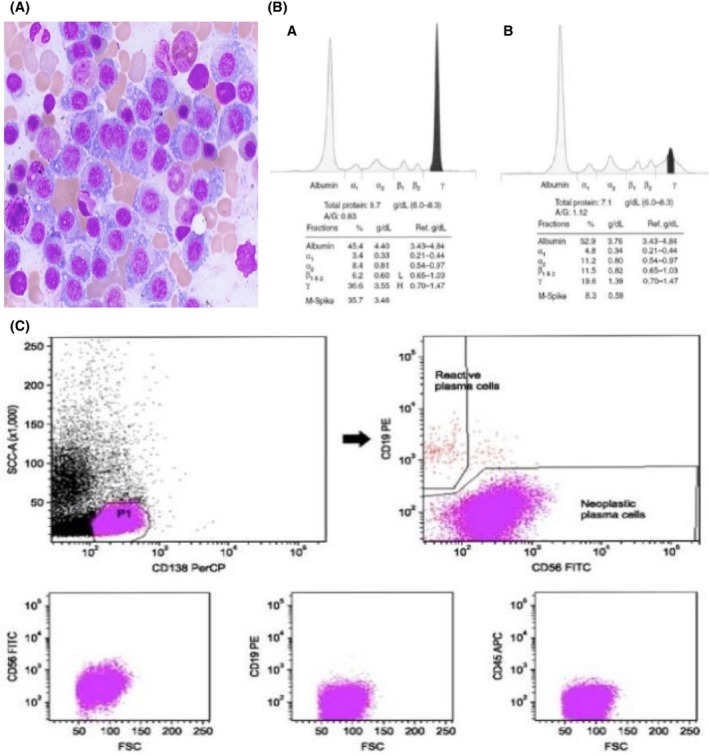
(A) Under *H&E*; 100×. Lesion shows cells with eccentric nucleus and prominent Golgi apparatus. (B) In situ hybridization showing serum monoclonal immunoglobulin A spikes of K‐light chains in both A and B sections. (C) IHC showing lesion cells staining CD 138 and CD 56 positive.

Histology of the L3 lesion revealed cells with eccentric nucleus and prominent Golgi apparatus (Figure 2A). Immunohistochemistry revealed cells stained positive for CD 138 and CD 56 (Figure 2B), and in situ hybridization showed a small serum monoclonal immunoglobulin A spike of K‐light chain (Figure 2C). WBC: 4.0/μL (<5.0/μL). LDH: 25 U/L (normal values <40.0 U/L). Renal function tests: Urea 6.0 mmol/L (2.5–8 mmol/L), creatinine: 70 mmol/L, (63–115 mmol/L), potassium: 4.0 meq/L (3.5–5.5 meq/L), sodium: 136 meq/L (135–155 meq/L), calcium: 6.0 mg/dL (8.5–10.5 mg/dL), and complete white blood cell count 5.0 (4.0–10.0 × 10^9^/L). Bone marrow evaluation was normal.

A diagnosis was made, and the child underwent radiation followed by systemic therapy causing reduction of L3 lesion.

### 2.3 Conclusion and results

This case involves 9‐year‐old male who has been diagnosed with multiple myeloma after thorough physical examination and investigation which have aided in the proper treatment and improving quality of life of our patient. The results have helped ruling out other possible causes of the child's back pain.

## 3 CASE DISCUSSION

### 3.1 Discussion and literature review

Plasma cell myeloma accounts for about 1% of all types of malignant diseases and slightly more than 10% of hematologic malignancies. The primary abnormality is a progressive and uncontrolled proliferation of immature and mature plasma cells in the bone marrow. This population of cells is believed to be monoclonal with a homogeneous production of immunoglobulin. The mean age at diagnosis has been reported at 62–64 years in several cases. Less than 1%–2% of patients are younger than 40 years of age at diagnosis.^1^

Our literature review shows some patients who have been previously described younger than age 30 years. One report describes a 13‐year‐old girl with breast tumors, IgA monoclonal gammopathy, and extensive bone marrow plasmacytosis. Another documents three young men, aged I7–22 years, with plasmacytomas and multiple lytic bone lesions; two of the three had an M‐protein. Bone marrow examination of all three revealed 5% morphologically normal plasma cells.^2^, ^3^

A survey reported from the National Cancer Institute (NIH) of 3815 cases of multiple myeloma showed seven cases occurring in patients under the age of 30 years; however, this study was only a statistical survey and did not contain any details of individual cases.^2^

The mean age of patients with solitary myeloma of bone is slightly younger than that of patients with plasma cell myeloma, being in the sixth decade.^4^

Another literature disclosed 24 cases in which plasma cell myeloma of bone occurred before the age of 30.^5^ In 17 out of 20 well‐documented cases were in young patients, the duration of survival was variable and ranged from 3 months to more than 10 years.^4^

Hewell et al. reported three cases with a relatively indolent course, whereas Clough et al. described aggressive disease in young female patients. Five patients survived for 5 years or more.^3^, ^4^, ^6^

Since the actual number of cases in young patients is so small, it is difficult to evaluate any difference in survival between younger and older patients. However, some seem to have more favorable prognosis in young patients than in older patients, because the 5‐year survival rate in a large series is 18%.^3^, ^7^

Of the reported cases are below the age of 30 years, serum or urine protein electrophoresis and/or immunocytochemical study for M‐protein were performed in 16 cases.^3^, ^8^

In 13 cases, M‐protein was demonstrated by serum or urine protein electrophoresis or both. In the remaining three cases, M‐protein could not be detected in the serum or urine, and therefore, these cases were considered to represent non‐secretory myeloma. In two of these three cases, tumor cells expressed monoclonal immunoglobulins in immunohistochemical studies. In seven cases, IgG paraprotein was expressed and in six IgA paraprotein. In one case, only few light chains were detected.^9^

Although the number of cases is small, IgA myeloma seems to be considerably more common in young patients, in contrast to a predominance of IgG myeloma in large series of all ages.^3^

Rapoport et al. suggested that in the younger group may differ clinically or biologically from that in the older group because of the predominance of IgA paraproteins. Anderson et al. stated that up to 15% of patients will have either a normal serum or a normal urine protein electrophoresis result, but in only 3 of 869 patients (0.35%) were both normal.

Plasma cell myeloma has been frequently diagnosed by the demonstration by laboratory studies such as bone marrow aspiration and serum and/or urine protein electrophoresis of the presence of M‐protein.^9^

However, it occasionally presents with features of a bone tumor.^10^ Plasma cell myeloma should thus be included in the list of differential diagnoses when solitary or multiple lytic lesions are encountered, even if these lesions occur in young persons.^10^

## 4 CONCLUSION

Plasma cell myeloma is extremely rare form of liquid tumor in the pediatric population, and it is important for any destructive bony lesion in a child to have appropriate work up for plasma cell myeloma.

## AUTHOR CONTRIBUTIONS

**Kato Ronald:** Conceptualization; formal analysis; investigation; methodology; resources; software; supervision; visualization; writing – original draft; writing – review and editing. **Ambaru Jacinta:** Formal analysis; investigation; project administration; writing – review and editing. **Ssebagala Umaru:** Methodology; project administration; resources; visualization.

## FUNDING INFORMATION

None for this publication.

## CONFLICT OF INTEREST STATEMENT

No conflict of interest.

## CONSENT

The consent was got and approved to publish this report from the patient's parents.

## Data Availability

The information that supports this study is available online through the link below https://www.ncbi.nlm.nih.gov/pmc/.
